# Early morphological change for predicting outcome in metastatic colorectal cancer after regorafenib

**DOI:** 10.18632/oncotarget.22807

**Published:** 2017-11-30

**Authors:** Hiroyuki Arai, Kunihisa Miyakawa, Tadamichi Denda, Takuro Mizukami, Yoshiki Horie, Naoki Izawa, Mami Hirakawa, Takashi Ogura, Takashi Tsuda, Yu Sunakawa, Takako Eguchi Nakajima

**Affiliations:** ^1^ Department of Clinical Oncology, St. Marianna University School of Medicine, Kawasaki, Japan; ^2^ Department of Radiology, St. Marianna University School of Medicine, Kawasaki, Japan; ^3^ Division of Gastroenterology, Chiba Cancer Center, Chiba, Japan

**Keywords:** colorectal cancer, regorafenib, morphological change, liver metastasis, lung metastasis

## Abstract

**Background and Objective:**

It is unclear whether early morphological change (EMC) is a predictive marker for regorafenib in metastatic colorectal cancer (mCRC). Therefore, the present study investigated whether EMC can predict the outcome of mCRC patients receiving regorafenib.

**Results:**

This study evaluated 68 patients. Among 52 patients with lung metastasis, 16 (31%) had cavity formation (CF). The median progression-free survival (PFS) and overall survival (OS) in patients with/without CF were 4.2/2.4 months (p<0.01) and 9.2/6.5 months (p=0.09), respectively. Among 45 patients with liver metastasis, 14 (31%) had active morphological response (MR). The median PFS and OS in patients with/without active MR were 5.3/2.4 months (p<0.01) and 13.6/6.9 months (p=0.02), respectively. Overall, 25 patients (37%) had EMC. The median PFS and OS in patients with/without EMC were 5.3/2.1 months (p<0.01) and 13.3/6.1 months (p<0.01), respectively.

**Materials and Methods:**

This retrospective study included mCRC patients with lung and/or liver metastases receiving regorafenib. CF in lung metastasis and MR in liver metastasis were evaluated at the first post-treatment computed tomography scan. EMC was determined as CF and/or active MR. We compared PFS and OS between patients with and those without EMC.

**Conclusions:**

EMC could be a useful predictive marker for regorafenib in mCRC.

## INTRODUCTION

Colorectal cancer is the third most common cancer and the fourth leading cause of cancer-related mortality worldwide [1]. Although the prognosis of patients with metastatic colorectal cancer (mCRC) has improved with the development of systemic chemotherapy, the median survival time is around 30 months, and this should be improved [2, 3]. Recently, two active agents (regorafenib and TAS-102) have been approved in Japan as salvage-line treatments for mCRC refractory to standard front-line chemotherapy.

Regorafenib is a multi-target tyrosine kinase inhibitor that targets angiogenic, stromal and oncogenic kinases and it was clearly demonstrated to prolong the survival duration of heavily treated mCRC patients when compared with placebo in two randomised phase III trials (CORRECT and CONCUR) [4, 5]. Regorafenib showed a disease control rate (DCR) of 41–51%. However, it caused some unfavourable side effects, such as hand-foot skin reaction, fatigue, hypertension and diarrhoea, which could worsen a patient's general condition and quality of life [4, 5]. Predictive markers for mCRC patients treated with regorafenib are desired, but such markers have not yet been elucidated [6].

The radiological changes with vascular endothelial growth factor (VEGF) pathway inhibitors have been suggested to be a surrogate for clinical activity [7, 8]. In colorectal cancer, Chun et al. reported that morphologic response (MR) based on computed tomography (CT) findings had a significant association with pathologic response and overall survival (OS) among patients with liver metastasis treated with bevacizumab-containing chemotherapy [9]. Furthermore, Ricotta et al. reported that cavity formation (CF) of lung metastasis on CT scans performed at the earliest post-baseline evaluation (at week 8) might predict OS and progression-free survival (PFS) in patients treated with regorafenib [10]. Therefore, early morphological change (EMC) might be a predictor of the outcome of patients receiving regorafenib. However, results have not been conclusive because of a small sample size and lack of validation.

Therefore, we conducted a multicenter retrospective study to investigate whether EMC can predict the outcome of mCRC patients receiving regorafenib.

## RESULTS

### Patient population and clinical outcomes

**Figure 1:**
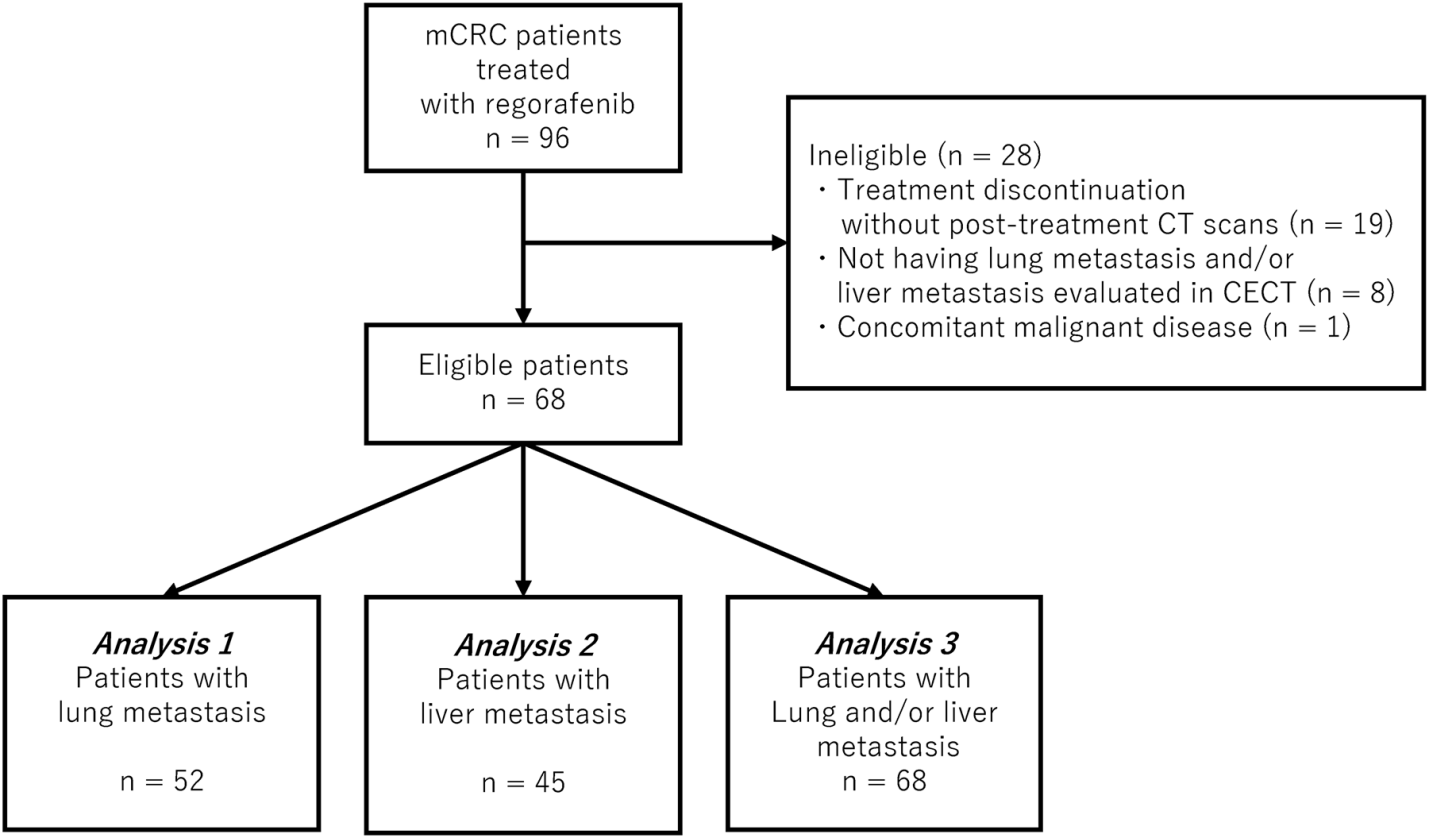
CONSORT diagram

The study evaluated 96 mCRC patients who received regorafenib. After excluding 28 patients who were not eligible (Figure 1), 68 patients were finally recruited in this study (analysis 1: 52 patients with lung metastasis, analysis 2: 45 patients with liver metastasis, and analysis 3: 68 patients with lung and/or liver metastases). The baseline characteristics are shown in Table 1. Almost all patients were refractory or intolerable to standard front-line chemotherapeutic agents (fluoropyrimidine, oxaliplatin and irinotecan; 100%, bevacizumab; 99%, anti-epidermal growth factor receptor (EGFR) antibody; 88%, KRAS or RAS wild-type tumour), whereas only 22% of the patients had previously received TAS-102. In each analysis, patient characteristics between the subgroup with and that without morphological change were almost well balanced. The median (range) period between the start of regorafenib therapy and the first post-treatment CT scan was 55 (20-133) days and this was comparable between the subgroup with and that without morphological change in each analysis.

**Table 1:** Patient characteristics

	Cohort 1Lung metastasis							Cohort 2Liver metastasis							Cohort 3Lung and/or liver metastasis						
	Total(N = 52)		CF+(N = 16)		CF-(N = 36)		Pvalue	Total(N =45)		MR+(N = 14)		MR-(N = 31)		Pvalue	Total(N =68)		EMC+(N = 25)		EMC-(N = 43)		Pvalue
	N	(%)	N	(%)	N	(%)		N	(%)	N	(%)	N	(%)		N	(%)	N	(%)	N	(%)	
Age (years)																					
Median	63		59		0.06	67		63		63		0.06	63		63		61		65		0.23
Range	41-80		43-70		41-80			40-79		44-74		40-79			40-80		43-74		40-80		
Sex																					
Male	25	(48)	5	(31)	20	(56)	0.14	28	(62)	10	(71)	18	(58)	0.51	37	(54)	13	(52)	24	(56)	0.80
Female	27	(52)	11	(69)	16	(44)		17	(38)	4	(29)	13	(42)		31	(46)	12	(48)	19	(44)	
PS																					
0	37	(71)	12	(75)	25	(69)	0.77	32	(71)	12	(86)	20	(65)	0.18	47	(69)	21	(84)	26	(60)	0.12
1	14	(27)	4	(25)	10	(28)		13	(29)	2	(14)	11	(35)		20	(29)	4	(16)	16	(37)	
2	1	(2)	0	(0)	1	(3)		0	(0)	0	(0)	0	(0)		1	(1)	0	(0)	1	(2)	
Histology																					
Tub1	20	(38)	7	(44)	13	(36)	0.25	13	(29)	7	(50)	6	(19)	0.18	23	(34)	11	(44)	12	(28)	0.47
Tub2	31	(60)	8	(50)	23	(64)		30	(67)	7	(50)	23	(74)		42	(62)	13	(52)	29	(67)	
Pap	0	(0)	0	(0)	0	(0)		1	(2)	0	(0)	1	(3)		1	(1)	0	(0)	1	(2)	
Unknown	1	(2)	1	(6)	0	(0)		1	(2)	0	(0)	1	(3)		2	(3)	1	(4)	1	(2)	
KRAS or RAS							0.13							0.34							>0.99
Wild type (WT)	29	(56)	6	(38)	23	(64)		27	(60)	10	(71)	17	(55)		41	(60)	15	(60)	26	(60)	
Mutant type (MT)	23	(44)	10	(63)	13	(36)		18	(40)	4	(29)	14	(45)		27	(40)	10	(40)	17	(40)	
Primary site																					
Colon	20	(38)	5	(31)	15	(42)	0.60	21	(47)	7	(50)	14	(45)	0.28	29	(43)	10	(40)	19	(44)	0.83
Rectum	28	(54)	9	(56)	19	(53)		23	(51)	6	(43)	17	(55)		35	(51)	13	(52)	22	(51)	
Cecum	4	(8)	2	(13)	2	(6)		1	(2)	1	(7)	0	(0)		4	(6)	2	(8)	2	(5)	
Primary side																					
Left	41	(79)	12	(75)	29	(81)	0.72	36	(80)	10	(71)	26	(84)	0.42	52	(76)	19	(76)	33	(77)	>0.99
Right	11	(21)	4	(25)	7	(19)		9	(20)	4	(29)	5	(16)		16	(24)	6	(24)	10	(23)	
Primary presence																					
Presence	9	(17)	3	(19)	6	(17)	>0.99	10	(22)	3	(21)	7	(23)	>0.99	11	(16)	5	(20)	6	(14)	0.52
Absence	43	(83)	13	(81)	30	(83)		35	(78)	11	(79)	24	(77)		57	(84)	20	(80)	37	(86)	
Disease status																					
Advanced	33	(64)	7	(44)	26	(72)	0.07	33	(73)	12	(86)	21	(68)	0.29	45	(66)	15	(60)	30	(70)	0.44
Recurrent	19	(36)	9	(56)	10	(28)		12	(27)	2	(14)	10	(32)		23	(34)	10	(40)	13	(30)	
No. of metastatic sites																					
1-2	24	(46)	9	(56)	15	(42)	0.38	26	(58)	10	(71)	16	(52)	0.33	37	(54)	15	(60)	22	(51)	0.61
3-5	28	(54)	7	(44)	21	(58)		19	(42)	4	(29)	15	(48)		31	(46)	10	(40)	21	(49)	
No. of prior regimens																					
2-3	39	(75)	12	(75)	27	(75)	>0.99	36	(80)	12	(86)	24	(77)	0.70	51	(75)	20	(80)	31	(72)	0.57
4-5	13	(25)	4	(25)	9	(25)		9	(20)	2	(14)	7	(23)		17	(25)	5	(20)	12	(28)	
Prior used agents																					
Fluoropyrimidine	52	(100)	16	(100)	36	(100)	>0.99	45	(100)	14	(100)	31	(100)	>0.99	68	(100)	25	(100)	43	(100)	>0.99
Oxaliplatin	52	(100)	16	(100)	36	(100)	>0.99	45	(100)	14	(100)	31	(100)	>0.99	68	(100)	25	(100)	43	(100)	>0.99
Irinotecan	52	(100)	16	(100)	36	(100)	>0.99	45	(100)	14	(100)	31	(100)	>0.99	68	(100)	25	(100)	43	(100)	>0.99
Bevacizumab	w51	(98)	16	(100)	35	(97)	>0.99	45	(100)	14	(100)	31	(100)	>0.99	67	(99)	25	(100)	42	(98)	>0.99
Anti-EGFR antibody (if KRAS or RAS WT)	26	(90)	5	(83)	21	(91)	0.52	22	(81)	7	(70)	15	(88)	0.28	36	(88)	11	(73)	25	(96)	0.05
TAS-102	11	(21)	4	(25)	7	(19)	0.72	8	(18)	4	(29)	4	(13)	0.23	15	(22)	6	(24)	9	(21)	0.77
Prior chemotherapy period (months)																					
Median	29.2		27.3		29.7		0.69	28.7		28.9		27.6		0.42	29.2		29.1		29.3		0.68
Range	6.8-85.7		6.8-75.4		7.3-85.7			6.8-85.7		18.3-76.1		6.8-85.7			6.8-85.7		6.8-76.1		7.3-85.7		
Starting dose of regorafenib																					
160mg/day	35	(67)	11	(69)	24	(67)	0.99	35	(78)	12	(86)	23	(74)	0.37	50	(74)	19	(76)	31	(72)	0.89
120mg/day	10	(19)	3	(19)	7	(19)		6	(13)	2	(14)	4	(13)		11	(16)	4	(16)	7	(16)	
80mg/day	7	(13)	2	(13)	5	(14)		4	(9)	0	(0)	4	(13)		7	(10)	2	(8)	5	(12)	
First CT evaluation (days)^*^																					
Median	56		57		56		0.42	55		52		56		0.51	55		55		55		0.88
Range	25-92		30-87		25-92			20-133		28-74		20-133			20-133		28-87		20-133		

^*^ Interval from the start of regorafenib to the first post-treatment CT scan.

**Figure 2:**
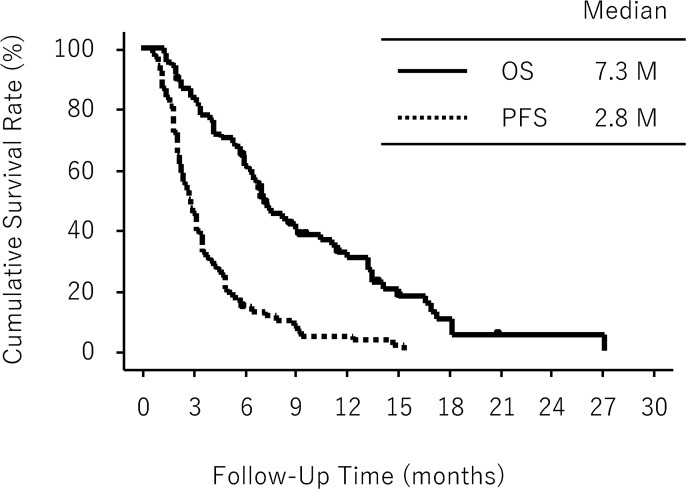
Overall survival (OS) and progression-free survival (PFS) in all eligible patients (n = 68)

Overall, median PFS and median OS were 2.8 and 7.3 months, respectively (Figure 2). Stable disease (SD) was noted in 34 patients (50%) and the remaining patients showed progressive disease (PD).

### Early morphological change

#### Analysis 1

**Figure 3:**
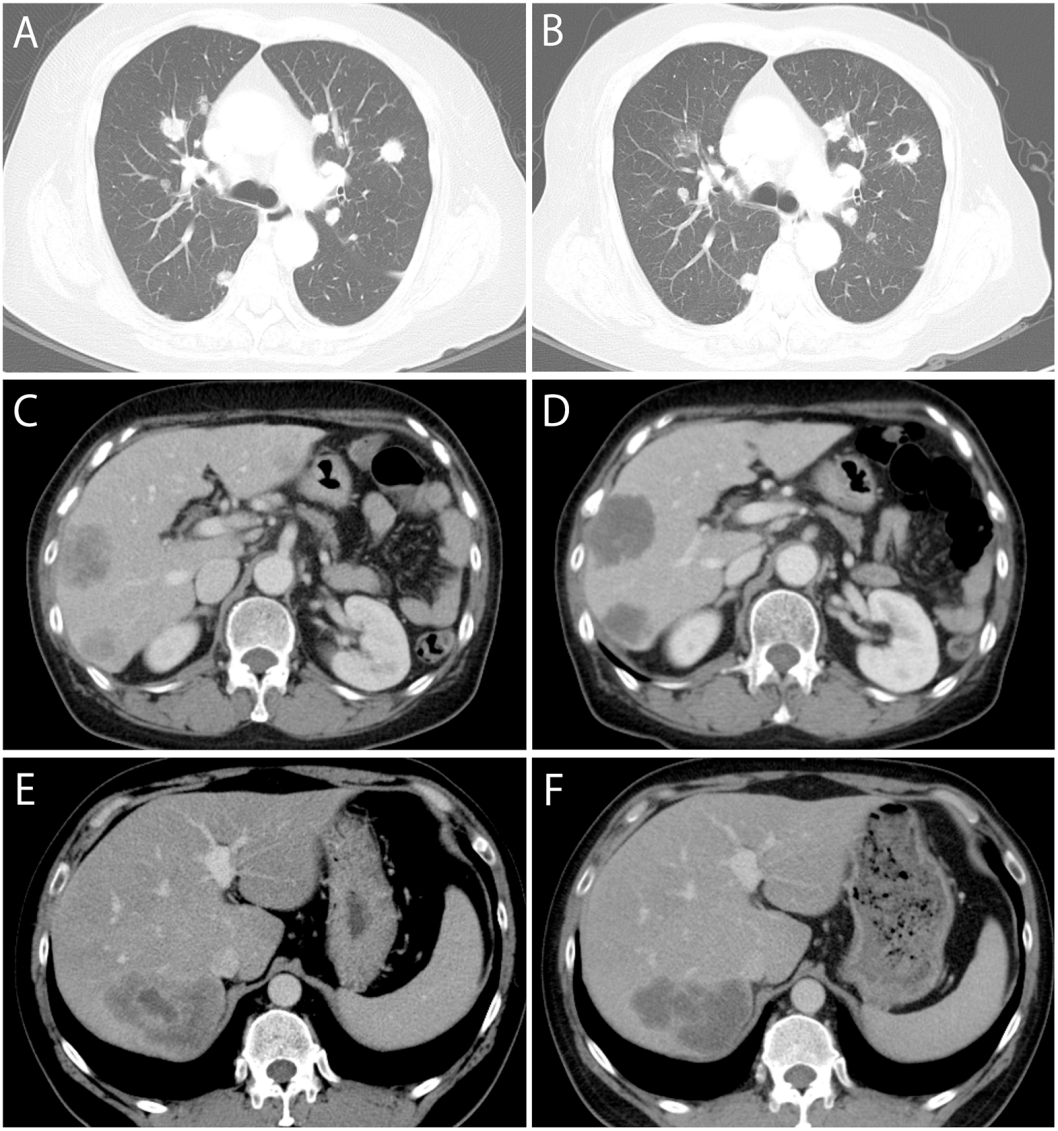
Exemplary CT images Upper case: A case with cavity formation (CF). Baseline **(A)** and first post-treatment **(B)** CT showed the emergence of CF. Middle case: A typical case (without discrepancy) with active morphological response (MR). Both evaluators classified liver metastases into group 3 at baseline CT **(C)** and group 2 at first post-treatment CT **(D)**. Therefore, this patient had ‘incomplete’ response. Lower case: A discrepant case with active MR. At baseline CT **(E)**, both evaluators classified liver metastases into group3. At first post-treatment CT **(F)**, there was discrepancy between two evaluators (group 3 and 2), and consensus review determined group2. Therefore, this patient had ‘incomplete’ response.

**Figure 4:**
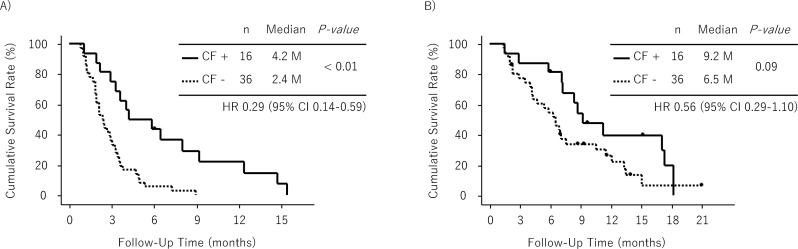
Analysis 1 (patients with lung metastasis) **(A)** Progression-free survival in patients with cavity formation (CF) and those without CF **(B)** Overall survival in patients with CF and those without CF.

Of the 52 patients in analysis 1, 16 (31%) had CF on the first post-treatment CT scan. There was no discrepancy among two evaluators with regard to the identification of CF. Among six patients with a pre-existing cavity at baseline, four experienced an increase in the pre-existing cavity. An exemplary CT image of CF is shown in Figure 3. PFS was significantly longer in patients with CF than in those without CF (hazard ratio [HR]: 0.29, 95% confidence interval [CI]: 0.14–0.59, p < 0.01, median PFS: 4.2 vs. 2.4 months) (Figure 4). OS was also longer in patients with CF than in those without CF; however, the difference was not significant (HR: 0.56, 95% CI: 0.29–1.10, p = 0.09, median OS: 9.2 vs. 6.5 months) (Figure 4). The DCR was significantly higher in patients with CF than in those without CF (81% vs. 36%, p < 0.01).

#### Analysis 2

Of the 45 patients in analysis 2, 39 and 6 patients were classified into groups 3 and 2, respectively, at the baseline CT scan. Among them, one patient in group 3 converted to group 1 (optimal response), 13 patients in group 3 converted to group 2 (incomplete response) and the rest did not show a group change (none) at the first post-treatment CT scan. Therefore, 14 (31%) patients had active MR. Discrepancy in grouping between the two evaluators occurred in 7 cases (16%) (5 cases included discrepancy about group 2 or 3 and 2 cases included discrepancy about group 1 or 2), but the discrepancy was resolved by consensus review. Exemplary CT images of active MR in both typical (without discrepancy) case and discrepant case are shown in Figure 3.

**Figure 5:**
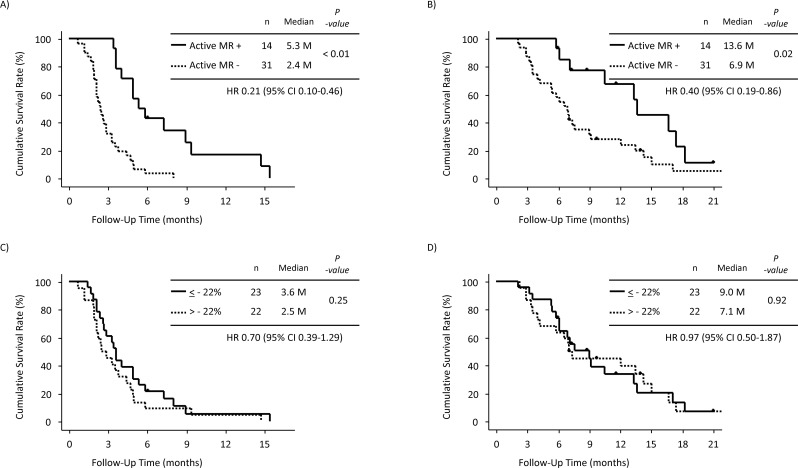
Analysis 2 (patients with liver metastasis) **(A)** Progression-free survival (PFS) in patients with active morphological response (MR) and those without active MR. **(B)** Overall survival (OS) in patients with active MR and those without active MR. **(C)** PFS in patients with an attenuation change of ≤ −22% and those with an attenuation change of > −22%. **(D)** OS in patients with an attenuation change of ≤ −22% and those with an attenuation change of > −22%.

PFS was significantly longer in patients with active MR than in those without active MR (HR: 0.21, 95% CI: 0.10–0.46, p < 0.01, median PFS: 5.3 vs. 2.4 months) (Figure 5). OS was also significantly longer in patients with active MR than in those without active MR (HR: 0.40, 95% CI: 0.19–0.86, p = 0.02, median OS: 13.6 vs. 6.9 months) (Figure 5). The DCR was significantly higher in patients with active MR than in those without active MR (100% vs. 39%, p < 0.01).

A total of 78 liver metastases from 45 patients were assessed for the attenuation value. The median attenuation value at baseline CT in the 45 patients was 63 Hounsfield unit (HU) (range, 26–90 HU), and the median attenuation change was −22% (range, −57 to 56). The DCR was equivalent between patients with an attenuation change of ≤ −22% (n = 23) and those with an attenuation change of > −22% (n = 22) (65% vs. 50%, p = 0.37). Both PFS and OS were also equivalent between patients with an attenuation change of ≤ −22% and those with an attenuation change of > −22% (Figure 5).

#### Analysis 3

**Figure 6:**
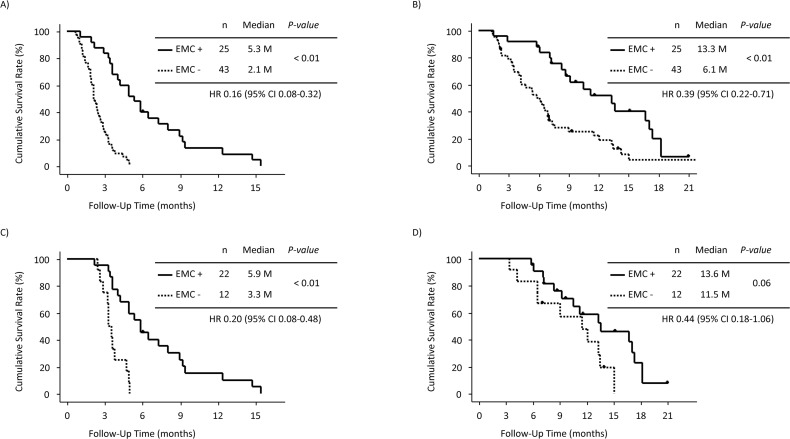
Analysis 3 (patients with lung and/or liver metastasis) **(A)** Progression-free survival (PFS) in patients with early morphological change (EMC) and those without EMC. **(B)** Overall survival (OS) in patients with EMC and those without EMC. **(C)** PFS in patients showing stable disease (SD) with EMC and those showing SD without EMC. **(D)** OS in patients showing SD with EMC and those showing SD without EMC.

Of the 68 patients in analysis 3, 25 (37%) had EMC. PFS was significantly longer in patients with EMC than in those without EMC (HR: 0.16, 95% CI: 0.08–0.32, p < 0.01, median PFS: 5.3 vs. 2.1 months) (Figure 6). OS was also significantly longer in patients with EMC than in those without EMC (HR: 0.39, 95% CI: 0.22–0.71, p < 0.01, median OS: 13.3 vs. 6.1 months) (Figure 6). The DCR was significantly higher in patients with EMC than in those without EMC (88% vs. 28%, p < 0.01).

Overall, among 34 patients (34/68, 50%) who showed SD, 22 (65%) had EMC. PFS was significantly longer in these 22 patients with EMC than in 12 patients without EMC (HR: 0.20, 95% CI: 0.08–0.48, p < 0.01, median PFS: 5.9 vs. 3.3 months) (Figure 6). OS was also longer in patients with EMC than in those without EMC; however, the difference was not significant (HR: 0.44, 95% CI: 0.18–1.06, p = 0.06, median OS: 13.6 vs. 11.5 months) (Figure 6).

## DISCUSSION

This is the first study to evaluate EMC for predicting the outcome of mCRC patients treated with regorafenib. We demonstrated that the frequency of EMC was 37% and that EMC could be a positive predictor for PFS, OS and the DCR.

CF in lung lesions has been reported to be an effect of angiogenesis inhibitors in lung cancer, with a frequency of 14–24% after treatment involving combination therapy or monotherapy with antiangiogenic agents [8, 11, 12]. Recently, Ricotta et al. reported about this morphological change in mCRC patients receiving regorafenib in a post-hoc analysis of the CORRECT study [13]. They showed that CF occurred with a high frequency (38.7%) and was associated with a lower rate of PD (CF+ vs CF−: 29.3% vs. 60.9%) at 8 weeks after starting regorafenib therapy. Our study demonstrated that CF occurred in 31% of patients at the first post-treatment CT scan and was associated with a higher DCR (CF+ vs. CF−: 81% vs. 36%), and these findings are consistent with the findings in the study by Ricotta et al. Furthermore, our results suggested that CF could predict better OS and PFS in patients receiving regorafenib, which has never been reported previously.

Anti-angiogenic agents are known to cause radiological change in liver lesions [9, 14]. This change can be described by the following two parameters: heterogeneity of attenuation and definition of the tumour-liver interface. Evaluation of MR is subjective with interpretation of these radiological parameters, and the frequency of discrepancy among evaluators in scoring MR has been previously reported to be 26% [9]. Discrepancy occurred in 18% of cases in the current study. On the other hand, change in the CT attenuation value is an objective parameter, and previous reports have shown that HU reduction after treatment with sorafenib or sunitinib was associated with longer PFS and greater tumour shrinkage in renal cell carcinoma [15, 16]. Therefore, we evaluated both MR and the change in the attenuation value for predicting the outcome in mCRC patients treated with regorafenib. Our results suggested that ‘optimal’ and ‘incomplete’ MR well predicted better OS, PFS and the DCR in comparison with ‘none’ MR. However, change in the CT attenuation value was not demonstrated to be a predictor of any outcome, which is consistent with the finding in a previous report [17]. We speculate that MR is more representable for the anti-angiogenic effect of regorafenib than CT attenuation change, because disease progression could also lead to HU reduction through lack of blood supply and central necrosis resulting from a rapid increase in tumour volume.

We evaluated EMC as a combination of CF and MR for the prediction of outcomes in patients with lung and/or liver metastases. Our result indicated that EMC could significantly predict longer OS and PFS, and a higher DCR. Interestingly, the prediction utility of EMC was also recognised in a limited population with SD according to RECIST.

It is important to carefully consider the risk–benefit balance of chemotherapy especially in salvage-line treatment for mCRC, because a patient's general condition tends to get rapidly worse and post-discontinuation treatment is limited. Unnecessary continuation of regorafenib should be avoided considering patient quality of life. Therefore, our results could be helpful for early decision-making on the discontinuation of the administration of regorafenib in clinical practice. If a patient has EMC at the first post-treatment CT scan, continuation of regorafenib can be encouraged because a longer PFS and OS could be expected. Conversely, if a patient does not have EMC, discontinuation of regorafenib and change to TAS-102 or best supportive care might be considered for a balance of toxicity, general condition, preference and other such factors.

The present study has some limitations. First, this was a retrospective study with a small sample size; another large cohort study may be required to validate and generalize our results. Second, the timing to evaluate EMC was not completely uniform. Although we usually conducted CT scan every 6-8 weeks, there were some cases with shorter or longer interval between treatment initiation and first post-treatment evaluation by physician's discretion. Last, MR is a subjective parameter, but frequency of discrepancy among evaluators was not different among studies.

In conclusion, both CF in lung metastasis and active MR in liver metastasis could predict good outcomes in mCRC patients treated with regorafenib. EMC, as a combination parameter of CF and active MR, may be an effective marker to encourage patients and physicians or to facilitate early decision-making for the discontinuation of regorafenib in clinical practice.

## MATERIALS AND METHODS

### Patients

We retrospectively reviewed the records of mCRC patients who received regorafenib between January 2011 and July 2016 at St. Marianna University School of Medicine or Chiba Cancer Center, Japan. All patient data were extracted from the hospital database. The eligibility criteria were: (1) histologically proven adenocarcinoma of the colon or rectum; (2) presence of lung and/or liver metastases; (3) absence of concomitant aggressive malignant diseases; (4) presence of a refractory or intolerant condition to fluoropyrimidine, oxaliplatin, irinotecan, anti-VEGF antibody and anti-EGFR antibody for KRAS (or RAS) wild-type tumour (including non-administration of agents because of comorbidity or refusal); (5) more than two prior regimens; (6) Eastern Cooperative Oncology Group performance status (ECOG PS) of 0–2; (7) CT assessment performed at baseline and after treatment (if only liver metastasis was present without lung metastasis, contrast-enhanced CT was required at baseline and after treatment). This study was approved by the Institutional Review Board of St. Marianna University School of Medicine and Chiba Cancer Center. All procedures were in accordance with the Helsinki Declaration. All patients provided written informed consent before treatment initiation.

### Chemotherapy

Patients orally received 160 mg of regorafenib once daily for the first 3 weeks of each 4-week session. Dose reduction, including starting dose, was allowed appropriately at the physician's discretion. Chemotherapy was repeated until disease progression, an unexpected serious adverse event, or patient refusal.

### Imaging analysis

In this study, we evaluated contrast-enhanced CT scans, with scanning in a single phase (portal venous phase) and a slice thickness of 5 mm, at both baseline and first evaluation after starting regorafenib therapy. An independent radiologist and independent oncologist blinded to the treatment outcome assessed EMC in the first post-treatment CT scan and compared the findings with those at baseline. EMC was defined as CF in lung metastasis and/or active MR in liver metastasis. CF was defined as the emergence of an air-filled cavity of ≥10% or increase in a pre-existing cavity, in at least one lung metastatic lesion. Active MR was defined as ‘optimal’ or ‘incomplete’ response, according to the criteria reported by Chun et al. in mCRC patients receiving bevacizumab [9]. Liver metastasis was classified into the following three groups: group 1, characterised by homogeneous attenuation with a thin, sharply defined tumour–liver interface; group 3, characterised by heterogeneous attenuation and a thick, poorly defined tumour–liver interface; and group 2, characterised by morphological features between those of groups 3 and 1. In the criteria presented by Chun et al., three types of MR were defined according to the pattern of group change from baseline to the first evaluation as follows: ‘optimal’ response if the group changed from 3 or 2 to 1; ‘incomplete’ response if the group changed from 3 to 2; and ‘none’ if the group did not change or increased. In cases with multiple liver metastases, MR was decided according to the dominant response pattern. If the grouping differed between the two evaluators, the group was determined by consensus review including careful discussion and fitting between them.

We also assessed the change in the CT attenuation value (Hounsfield unit [HU]) for liver metastasis between baseline and the first post-treatment assessment. Attenuation change was calculated as follows: attenuation change (%) = (HU_first post-treatment CT_–HU_baseline CT_) / HU_baseline CT_ × 100. For measurement in HU, a round region of interest (ROI) was placed across the maximum area in each liver metastasis, and the mean attenuation value of the ROI was measured. If a patient had multiple liver metastases, two lesions were selected in decreasing order of size, and the mean attenuation value was calculated in each patient.

### Statistical analysis

We performed the following analyses: analysis 1 (included patients with lung metastasis), analysis 2 (included those with liver metastasis) and analysis 3 (included those with lung and/or liver metastases). Patient demographics were summarised by descriptive statistics. These demographics were compared between patients with and those without morphological change using Fisher's exact test (binary variables) or the chi-square test (ternary variables) for categorical variables and the Mann–Whitney *U* test for continuous variables.

PFS was defined as the time from initiation of regorafenib to disease progression or death from any cause, and OS was defined as the time from initiation of regorafenib to death from any cause. The cut-off date of the observation period was December 31, 2016. Patients who had no events during the observation period were censored at the last follow-up date. Both PFS and OS were compared between patients with and those without morphological change, using the log-rank test. Cox proportional hazards model was used to calculate hazard ratio (HR) and confidence interval (CI).

Tumour response was assessed using the Response Evaluation Criteria in Solid Tumours (RECIST) version 1.1. Confirmation of SD required at least a 6-week interval from the start of treatment. The DCR was defined as the proportion of patients with the best response with regard to complete response, partial response, or SD. The DCR was compared between patients with and those without morphological change using Fisher's exact test.

In all analyses, a two-sided p-value <0.05 was considered statistically significant. All statistical analyses were performed using StatView ver 5.0 software (SAS Institute, Cary, NC).

## Abbreviations

EMC: early morphological change
CF: cavity formation
MR: morphological response
mCRC: metastatic colorectal cancer
CT: computed tomography
HU: Hounsfield unit
RECIST: Response Evaluation Criteria in Solid Tumours

## References

[1] International Agency for Research on Cancer GLOBOCAN 2012: cancer incidence and mortality worldwide. http://globocan.iarc.fr/Default.aspx.

[2] Yamazaki K, Nagase M, Tamagawa H, Ueda S, Tamura T, Murata K, Eguchi Nakajima T, Baba E, Tsuda M, Moriwaki T, Esaki T, Tsuji Y, Muro K (2016). Randomized phase III study of bevacizumab plus FOLFIRI and bevacizumab plus mFOLFOX6 as first-line treatment for patients with metastatic colorectal cancer (WJOG4407G). Ann Oncol.

[3] Stintzing S, Modest DP, Rossius L, Lerch MM, von Weikersthal LF, Decker T, Kiani A, Vehling-Kaiser U, Al-Batran SE, Heintges T, Lerchenmuller C, Kahl C, Seipelt G (2016). FOLFIRI plus cetuximab versus FOLFIRI plus bevacizumab for metastatic colorectal cancer (FIRE-3): a post-hoc analysis of tumour dynamics in the final RAS wild-type subgroup of this randomised open-label phase 3 trial. Lancet Oncol.

[4] Grothey A, Van Cutsem E, Sobrero A, Siena S, Falcone A, Ychou M, Humblet Y, Bouche O, Mineur L, Barone C, Adenis A, Tabernero J, Yoshino T (2013). Regorafenib monotherapy for previously treated metastatic colorectal cancer (CORRECT): an international, multicentre, randomised, placebo-controlled, phase 3 trial. Lancet.

[5] Li J, Qin S, Xu R, Yau TC, Ma B, Pan H, Xu J, Bai Y, Chi Y, Wang L, Yeh KH, Bi F, Cheng Y (2015). Regorafenib plus best supportive care versus placebo plus best supportive care in Asian patients with previously treated metastatic colorectal cancer (CONCUR): a randomised, double-blind, placebo-controlled, phase 3 trial. Lancet Oncol.

[6] Tabernero J, Lenz HJ, Siena S, Sobrero A, Falcone A, Ychou M, Humblet Y, Bouche O, Mineur L, Barone C, Adenis A, Yoshino T, Goldberg RM (2015). Analysis of circulating DNA and protein biomarkers to predict the clinical activity of regorafenib and assess prognosis in patients with metastatic colorectal cancer: a retrospective, exploratory analysis of the CORRECT trial. Lancet Oncol.

[7] O’Connor JP, Jayson GC (2012). Do imaging biomarkers relate to outcome in patients treated with VEGF inhibitors?. Clin Cancer Res.

[8] Crabb SJ, Patsios D, Sauerbrei E, Ellis PM, Arnold A, Goss G, Leighl NB, Shepherd FA, Powers J, Seymour L, Laurie SA (2009). Tumor cavitation: impact on objective response evaluation in trials of angiogenesis inhibitors in non-small-cell lung cancer. J Clin Oncol.

[9] Chun YS, Vauthey JN, Boonsirikamchai P, Maru DM, Kopetz S, Palavecino M, Curley SA, Abdalla EK, Kaur H, Charnsangavej C, Loyer EM (2009). Association of computed tomography morphologic criteria with pathologic response and survival in patients treated with bevacizumab for colorectal liver metastases. JAMA.

[10] Ricotta R, Sartore-Bianchi A, Verrioli A, Vanzulli A, Siena S (2013). Regorafenib for metastatic colorectal cancer. Lancet.

[11] Nishino M, Cryer SK, Okajima Y, Sholl LM, Hatabu H, Rabin MS, Jackman DM, Johnson BE (2012). Tumoral cavitation in patients with non-small-cell lung cancer treated with antiangiogenic therapy using bevacizumab. Cancer Imaging.

[12] Marom EM, Martinez CH, Truong MT, Lei X, Sabloff BS, Munden RF, Gladish GW, Herbst RS, Morice RC, Stewart DJ, Jimenez CA, Blumenschein GR, Onn A (2008). Tumor cavitation during therapy with antiangiogenesis agents in patients with lung cancer. J Thorac Oncol.

[13] Ricotta R, Verrioli A, Ghezzi S, Grothey A, Cremolini C, Argiles G, Adenis A, Ychou M, Barone C, Bouchet O, Humblet Y, Mineur L, Sobrero A (2015). Cavitation of lung metastases induced by regorafenib in patients with colorectal carcinoma: Data from the phase III CORRECT study. Eur Cancer Congr.

[14] Boonsirikamchai P, Asran MA, Maru DM, Vauthey JN, Kaur H, Kopetz S, Loyer EM (2011). CT findings of response and recurrence, independent of change in tumor size, in colorectal liver metastasis treated with bevacizumab. AJR Am J Roentgenol.

[15] Smith AD, Lieber ML, Shah SN (2010). Assessing tumor response and detecting recurrence in metastatic renal cell carcinoma on targeted therapy: importance of size and attenuation on contrast-enhanced CT. AJR Am J Roentgenol.

[16] Cowey CL, Fielding JR, Rathmell WK (2010). The loss of radiographic enhancement in primary renal cell carcinoma tumors following multitargeted receptor tyrosine kinase therapy is an additional indicator of response. Urology.

[17] Lim Y, Han SW, Yoon JH, Lee JM, Lee JM, Paeng JC, Won JK, Kang GH, Jeong SY, Park KJ, Lee KH, Kim JH, Kim TY (2015). Clinical Implication of Anti-Angiogenic Effect of Regorafenib in Metastatic Colorectal Cancer. PLoS One.

